# A rare case of obesity. Can it be Bardet‐Biedl Syndrome?

**DOI:** 10.1002/ccr3.2356

**Published:** 2019-08-04

**Authors:** Sandeep Shrestha, Nagendra Chaudhary

**Affiliations:** ^1^ Department of Pediatrics Universal College of Medical Sciences Bhairahawa Nepal

**Keywords:** Bardet‐Biedl syndrome, hypothyroidism, obesity, poor vision

## Abstract

Bardet‐Biedl Syndrome (BBS) is a rare autosomal recessive disorder with a wide spectrum of clinical manifestations like retinal dystrophy, obesity, polydactyly, mental retardation, hypogonadism, and renal dysfunction. We report a case of 14‐year‐old obese boy with poor scholastic performances, hypothyroidism, and poor vision diagnosed as BBS.

## 1 INTRODUCTION

Bardet‐Biedl Syndrome (BBS) is a rare autosomal recessive genetic disorder with wide spectrum of clinical presentation. It has a multisystem involvement with variable clinical manifestations but the cardinal features include progressive retinal dystrophy, central obesity, postaxial polydactyly, hypogonadism, learning disabilities, and renal dysfunction.^1^ Other minor defects include speech delay, developmental delay, diabetes mellitus, dental anomalies, sensorineural deafness, ataxia, congenital heart disease, and hepatic fibrosis. Four out of six major criteria are required to make a clinical diagnosis of BBS (Table 1).^2^ Genetic analysis is needed for definitive diagnosis which shows mutation of BBS gene.^3^

**Table 1:** Showing primary and secondary features

Primary features	Secondary features
Cone‐rod dystrophy	Speech disorder/delay
Polydactyly	Strabismus/cataracts/astigmatism
Obesity	Brachydactyly/syndactyly
Learning disabilities	Developmental delay
Hypogonadism in males	Polyuria/polydipsia (nephrogenic diabetes insipidus)
Renal anomalies	Ataxia/poor coordination/imbalance
	Mild spasticity
	Diabetes mellitus
	Dental crowding/hypodontia/small roots/high‐arched palate
	Left ventricular hypertrophy/congenital heart disease
	Hepatic fibrosis

## 2 CASE REPORT

**Figure 1:**
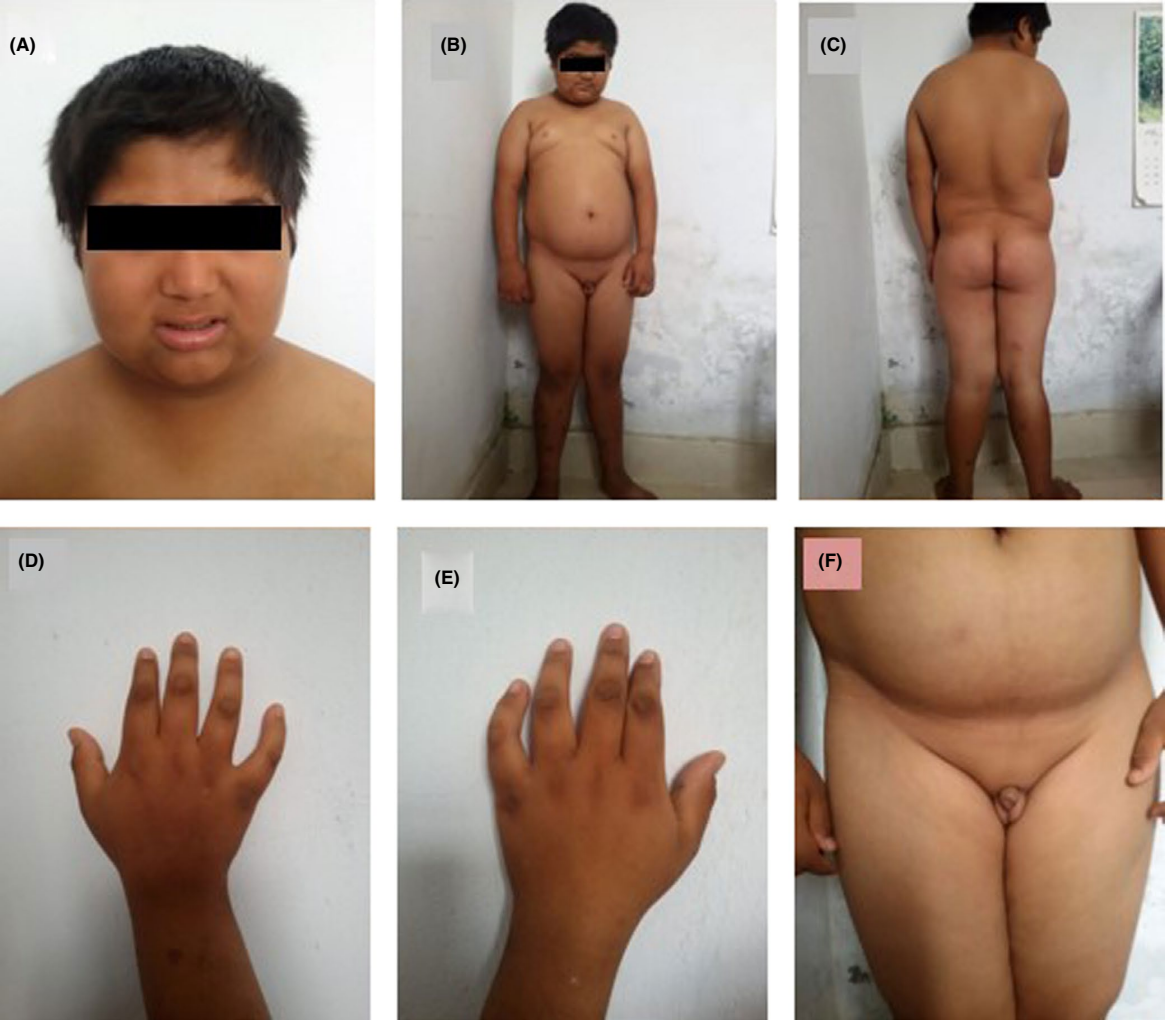
Physical features in the child with Bardet‐Biedl Syndrome (A, typical facial appearance “broad facies, flat nasal bridge, long philtrum, and thin upper lip”; B and C, characteristic truncal obesity; D and E, short and broad fingers [no polydactyly in this case]; F, hypogonadism with micropenis)

A 14‐year‐old male child, product of nonconsanguineous parents, presented to pediatric outpatient department with the chief complains of excessive weight gain since 8 years of age. On further inquiry, the child was found to have poor scholastic performance and skills. There was no family history of obesity. Antenatal, natal, and postnatal periods were uneventful. Developmental milestones were attained as per age except for his intelligence and scholastic performances. Other siblings were normal. On examination, his height was 144 cm (*Z* score = −2.4) and weight 65 kg (>85th Centile) with BMI of 31.3 kg/m^2^ (98.6 percentile). Blood pressure was at 75th centile for his age. Genital examination showed bilateral undescended testis with micropenis (Stretched Penile Length‐1.5 cm; Figure 1).

**Figure 2:**
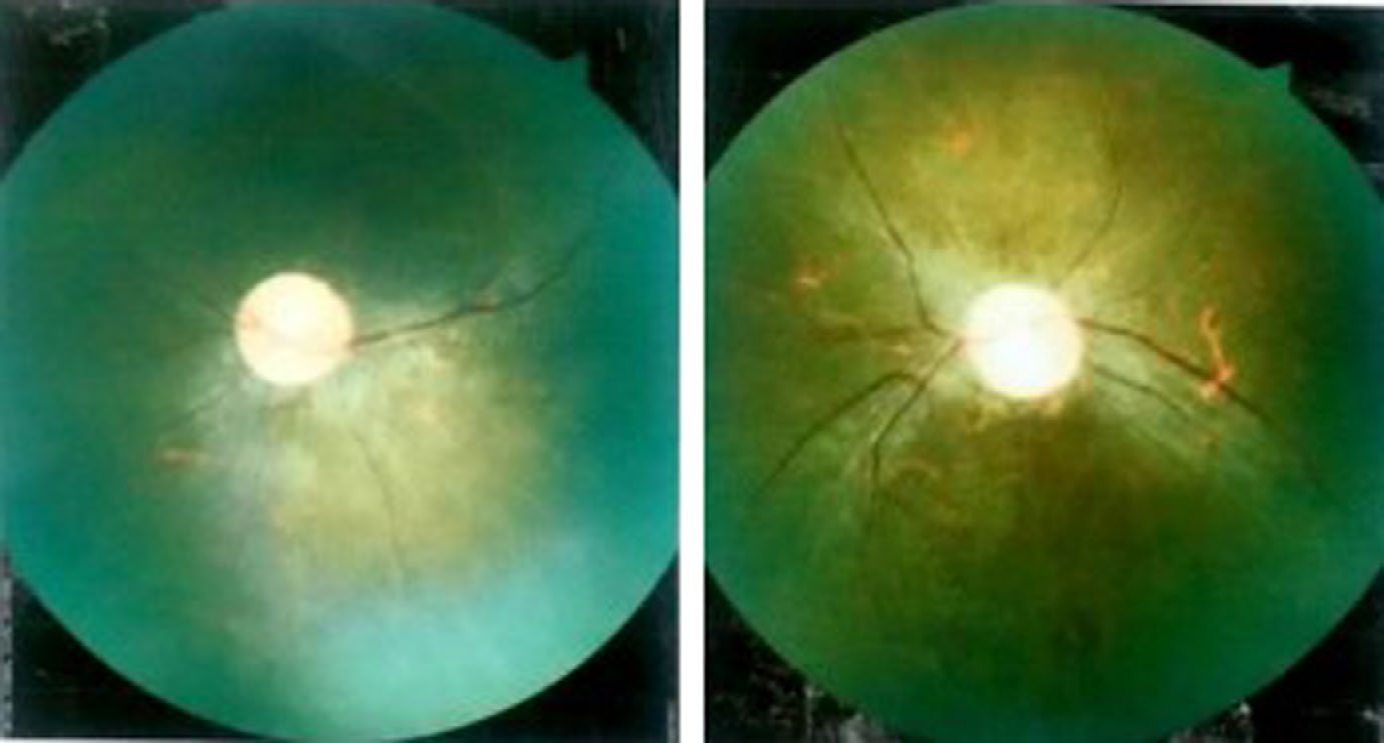
Fundoscopic examination of right and left eyes showing optic atrophy, attenuated blood vessels, and bony spicules suggestive of retinitis pigmentosa

Respiratory and cardiac systems examination was normal. Workup for short stature and obesity was considered. Serum cortisol was normal. Thyroid function test revealed raised thyroid‐stimulating hormone (TSH) level (TSH‐ 8.710 uIU/mL) with near normal T_3_ and T_4_ levels (T_3_‐ 90 ng/dL, T_4_‐ 5 μg/dL). Serum testosterone was low (0.025 ng/dL), whereas serum luteinizing hormone (LH) and follicle‐stimulating hormone (FSH) were within normal ranges for age. Growth hormone was within the normal limits. Chest x‐ray, blood sugar, routine urine microscopic examination, and renal function test were normal. Ultrasound scan of abdomen and magnetic resonance imaging of brain were normal. Short stature with poor scholastic performance and abnormal thyroid function test was attributed due to hypothyroidism. Child was then started on oral l‐thyroxine, initially on 25 μg/d and was increased to 50 μg/d based on the TSH levels. Repeat thyroid function test after 1 month of l‐thyroxine (50 μg/d) was in the normal range. Child was followed up every 3‐4 months but did not have any improvement in his weight and school performance. He also started having visual symptoms after 2 years of follow‐up (at the age of 16 years). Child started complaining of difficulty in seeing at night which was progressive in nature. Fundoscopic examination showed findings typical of retinitis pigmentosa (Figure 2).

The child did not have any limb deformities. Based on the above clinical findings—obesity, cone‐rod dystrophy, poor scholastic performance, and hypogonadism, child was diagnosed as a case of BBS. Consent for genetic analysis was obtained from the parents.

### 2.1 Genetic analysis

Venous blood of the child was drawn by venipuncture which was collected in acid citrate dextrose vacutainers. DNA extraction was done by standard phenol‐chloroform extraction procedure.^4^

Exome data analysis showed previously reported missense mutation 442G>A (p.Asp148Asn) in the BBS 1 gene and on segregation analysis; we found mutation on both the alleles (homozygous). No other mutation was detected. Genetic counseling was done to the parents. Low‐calorie diet with regular exercise was advised for obesity.

## 3 DISCUSSION

The estimated prevalence of BBS varies from 1:160 000 in Northern European population to 1:135 00 in isolated communities in Kuwait.^5^ In 1886, Laurence and Moon^6^ first described this syndrome comprising of retinal dystrophy, obesity, spastic paraparesis, and cognitive defect. In 1920, Bardet and Biedel reported similar cases with additional polydactyly, and this was termed as Laurence‐Moon‐Bardet‐Biedl syndrome.^7^ Now, BBS is considered as a separate entity with modified diagnostic criteria consisting of primary and secondary features (Table 1). Four primary features or three primary plus two secondary features are required to diagnose BBS clinically.^2^ Our case fulfilled four primary features for the diagnosis of BBS.

Retinal dystrophy is the most common major manifestation present in BBS. Among the clinical features, atypical retinal degeneration is the most common clinical presentation. Patient usually presents with night blindness which gradually progresses to blindness.^8^ The mean age at which patient complains of night blindness is 8.5 years (range 1‐34 years), and by 15.5 years (range 8‐43 years), most of them are registered blind.^2^ In our case, night blindness was noticed at the age of 16 years. Obesity is present in 72%‐92% of cases, whereas hypogonadism is present in 59%‐98% of BBS cases. Learning difficulty is found to be present in 50%‐61% of cases.^2^ Our case also had obesity, hypogonadism (micropenis and low testosterone levels), and learning difficulties. Polydactyly (postaxial) is noted in 63%‐81% of BBS patients. However, our patient did not have polydactyly or any other limb anomalies.

Our case is interesting as the child initially had only obesity and raised TSH levels along with poor academic performances for which he was diagnosed as a case of hypothyroidism. This case report emphasizes that despite abnormal thyroid function test in a child with obesity, one should consider other possible etiologies.

Renal anomalies are seen in 20%‐53% of children with BBS, the most common presentation being structural anomalies (parenchymal cysts, calyceal cysts, vesico‐ureteric reflux, and horseshoe kidney).^2^ Some may manifest as chronic kidney disease and abnormal urine analysis.^9^ Ultrasound scan of abdomen of our patient did not show any renal anomalies. Urine analysis and renal function test were also normal. Although cardiovascular examination and blood pressure were normal, echocardiographic examination showed mild concentric hypertrophy of left ventricle in our case.

Definite diagnosis of BBS requires genetic analysis which shows mutation of BBS gene. Till date, 21 BBS genes have been identified.^10^ Mutation in BBS‐1 gene alone accounts for 70%‐80% of all BBS cases.^3^, ^11^ In our case, genetic analysis showed homozygous missense mutation in the BBS‐1 gene that has been reported earlier.^12^

## 4 CONCLUSION

Bardet‐Biedl Syndrome, although a rare condition, should be sought in a child with obesity, poor scholastic performances, and hypothyroidism. Proper history, detailed clinical examination, and ocular examination help in the clinical diagnosis of BBS in resource poor countries even if genetic analysis is not available. Definitive diagnosis is done by gene mutation studies.

## CONFLICT OF INTEREST

None declared.

## AUTHOR CONTRIBUTIONS

SS and NC: equally involved in drafting, literature search, and writing of the paper.

## CONSENT

Both verbal and written consents were obtained from the parents regarding the publication of the case and photographs.
